# Intensive Care Usage by HIV-Positive Patients in the HAART Era

**DOI:** 10.1155/2011/847835

**Published:** 2011-10-30

**Authors:** L. Turtle, R. Vyakernam, A. Menon-Johansson, M. R. Nelson, N. Soni

**Affiliations:** ^1^Tropical and Infectious Diseases Unit, Royal Liverpool University Hospital, Prescot Street, Liverpool, L7 8XP, UK; ^2^Magill Department of Anaesthesia Intensive Care and Pain Management, Chelsea and Westminster Hospital, 369 Fulham Road, London, SW10 9NH, UK; ^3^Department of HIV/GUM, Chelsea and Westminster Hospital, 369 Fulham Road, London, SW10 9NH, UK

## Abstract

In the 1980s the outlook for patients with the acquired immunodeficiency syndrome (AIDS) and critical illness was poor. Since then several studies of outcome of HIV+ patients on ICU have shown improving prognosis, with anti-retroviral therapy playing a large part. We retrospectively examined intensive care (ICU) admissions in a large HIV unit in London. Between April 2001 and April 2006 43 patients were admitted to the ICU. The mean age of patients was 44 years and 74% were male. Fifty-six percent of admissions were receiving anti-retroviral therapy and 44% had an AIDS defining diagnosis. The median CD4 count was 128 cells/mL and the median APACHE II score was 21. The commonest diagnostic ICU admission category was respiratory disease. This group experienced higher mortality despite slightly lower APACHE II scores, though this did not achieve statistical significance. The follow up period was one year or until April 2007, when data were censored. ICU mortality was 33%, in hospital mortality was 51% and overall mortality at the end of the study period was 67%. Median survival was 1008 days. The CD4 count did not predict long-term survival, although the sample size was too small for this to be conclusive.

## 1. Introduction

In the 1980s, the outlook for patients with the acquired immunodeficiency syndrome (AIDS) and critical illness was poor [1, 2], and intensive care for these patients was felt inappropriate [3, 4]. Most studies showed the worse survival the lower the CD4 count [5–10]. The prognosis of HIV-infection has improved dramatically with highly active antiretroviral therapy (HAART) [11–13]. Survival of HIV+ individuals admitted to intensive care has now improved, the case mix has changed and CD4 count at admission relates less to outcome [14–21]. We, in combination with others, have previously published our intensive care experience with HIV infected patients from the mid 1990s [7]. Survival was shorter with CD4 count <100 cells/mL. Few of the published studies, however, have followed up patients long term [16, 22], and these only recruited up until 1999.

In recent years, the long-term prognosis of HIV+ individuals has continued to improve. With longer survival and more patients on treatment, the cohort of HIV+ patients has increased in size. In the UK, still one-third of patients are diagnosed with advanced HIV disease (CD4 count <200 cells/mm^3^). Therefore, we undertook a service review to examine usage of the ICU facility in our hospital by HIV+ patients. We have investigated the numbers of admissions, case mix, and long-term survival following intensive care unit (ICU) admission during recent years in a large London unit.

## 2. Methods

The Chelsea and Westminster Hospital in South West London provides care for over 5000 HIV-infected individuals, one of the largest cohorts in the UK. We conducted a retrospective review of electronic ICU records of known HIV+ patients of our hospital who were admitted to the ICU between April 1st, 2001 and April 1st, 2006. All of the patients included in this study were already under the care of the HIV/GUM department for their HIV disease. The hospital ethics committee was consulted and deemed formal ethical review unnecessary. Data collected were age at admission, sex, race, risk factor for HIV acquisition, receipt of anti-retroviral therapy, admission diagnosis, reason for ICU admission, CD4 count, HIV viral load, acute physiology and chronic health evaluation II (APACHE II) score, length of stay, organs systems supported, and outcome. Admissions were defined as AIDS-related if prompted by an AIDS defining illness [23]. Standard definitions were used for sepsis and septic shock [24]. Followup lasted until April 1st, 2007, a minimum followup period of one year for all patients.

### 2.1. Data Analysis

Cox regression was used to correlate variables of interest with outcome. Kaplan-Meier analysis and log rank test were used to examine long-term survival. The Wilcoxon rank sum test was used to compare CD4 counts between groups. Stata 10 and *r* software were used for analysis. A significance level of 0.05 was used throughout.

## 3. Results

### 3.1. Descriptive Analysis

During the study period, 43 patients were admitted to the ICU, each on one occasion. All patients were known to be HIV+ at the time of ICU admission, there were no diagnoses made on the ICU during the study period. Characteristics of the patients in this study are shown in Table 1.

### 3.2. Reason for Admission to ICU and Usage

The reasons for admission to ICU by category and subsequent outcome are shown in Table 2. All of the patients in this study had only one admission to ICU. The mean length of ICU stay was 10 days (median 6 days; range 1 to 47).

Thirty-three patients (78.7%) had respiratory support, 26 (62%) underwent tracheal intubation and mechanical ventilation, and seven (16.7%) had noninvasive ventilation. Twenty-three patients (56%) had cardiovascular support with inotropes (data missing for two patients). Eleven patients (26.8%) had renal replacement therapy (data missing for two patients).

### 3.3. Diagnosis

A wide variety of diagnoses were made (Table 3). Seven patients had more than one diagnosis made (not including patients with organ failure as part of a sepsis syndrome or neutropaenic sepsis occurring on the background of chemotherapy for malignancy). These were Castleman's disease/PCP (at presentation), Burkitt's lymphoma/pneumonia (at presentation), PCP/pulmonary oedema, pneumonia/pontine haemorrhage, tuberculous arthritis/renal failure (unexplained), anal carcinoma/renal failure (drug related), and multiple myeloma/non-Hodgkin's lymphoma.

Nineteen admissions (44%) were for AIDS-related diagnoses (according to 1993 CDC criteria). However, 31 admissions (72%) in total were for HIV-related diagnoses, this includes the 19 AIDS-related admissions plus further 12 patients with serious infections but CD4 counts greater than 200. Twelve patients were admitted to ICU for reasons not directly related to HIV infection. These were pneumothorax/threatened airway, pancreatitis (2 patients), GI bleed (2 patients), liver failure, renal failure (2 patients), status epilepticus, deliberate drug overdose, adhesional bowel obstruction on a background of ulcerative colitis, and neutropaenic sepsis related to chemotherapy for anal carcinoma (considered not related to HIV).

### 3.4. CD4 Count and Antiretroviral Therapy

The median CD4 count was 128 cells/mL (range 4 to 958). In the 19 patients (44%) who were admitted to ICU after diagnosis of an AIDS-defining illness, median CD4 count was lower; 58 versus 210 cells/mL (*P* < 0.001). Median CD4 count in patients with any HIV-related admission (not only AIDS) was lower compared with patients admitted for reasons unrelated to HIV infection (100 versus 242 cells/mL, *P* < 0.001).

Twenty-four patients of 37 patients for whom data were available were on HAART before ICU admission. Median CD4 counts in patients on HAART and not on HAART were 188 cells/mL and 89 cells/mL, respectively. Of the 24 patients on HAART, 16 (66.6%) were virologically suppressed. Eight patients had detectable HIV RNA in plasma; four of these had a viral load less than 400 copies/mL.

### 3.5. Survival

Mortality rates throughout the study period are shown in Table 2. Fourteen patients died on the ICU; the median time to death on ICU was 8.5 days (mean 9.8 days, IQR 2 to 16 days; range 1 to 23 days). Eight patients survived after ICU admission but died in hospital; median time to death for this group was 30 days from the date of ICU admission (mean 37.1 days; range 3 to 77). Twenty-two patients (51%) died in hospital. Median time to death of those who survived to hospital discharge but died during followup (seven patients) was 352 days from ICU admission (mean 295.7 days; range 81 to 565). Median time to death from ICU admission for nonsurvivors was 20 days (mean 86.3 days).

Mortality was higher than average in patients with a respiratory diagnosis (Table 2) although the median APACHE II score was lower in the respiratory group (17 versus 26). Adjustment for this, however, showed no significant difference in mortality between the two groups. Mortality among patients requiring invasive ventilation was slightly, though not significantly, higher than total mortality (*P* = 0.108).

**Figure 1:**
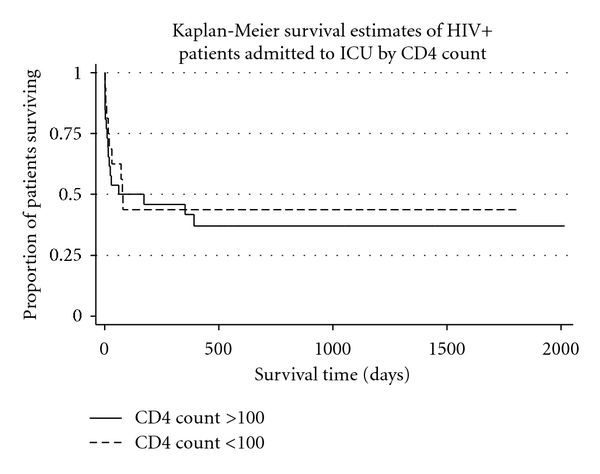
Survival of HIV+ patients admitted to ICU by CD4 count category greater or less than 100. *P* = 0.56 by log rank test. Analysis using a CD4 cut-off of 200 or 50 gave a similar result.

We did not observe any association between CD4 count and survival in this population (*P* = 0.77).  Figure 1 shows Kaplan-Meier analysis of survival for patients with a CD4 count greater than or less than 100. There was no difference in long-term survival between patients with CD4 counts above or below 100 cells/mL (log rank test, *P* = 0.56). Analysis using CD4 count cut-offs of 50 and 200 cells/mL gave similar results.

There was a nonsignificant trend to increasing mortality with increasing APACHE II score, hazard ratio 1.04 per point increase in APACHE II score, *P* = 0.056 (a doubling of mortality with 17 point increase in APACHE II score). No other variables were significantly associated with mortality; therefore, multivariate analysis was not performed. 

The median followup for the study was 1008 days (range 1–2015 days). Overall, 29 (67.4%) patients died during the study period. Median survival in those who died was 20 days after ICU admission (range 1–565 days). Twenty-six of the 29 patients who died had died by one year of followup. Although early mortality was high, all patients that survived more than two years after the date of ICU admission were alive at the end of the study period.

## 4. Discussion

It is striking that, despite coming from a large unit, the number of patients admitted to ICU during a five-year period was low, only 43. Because of this small number, it is difficult to make generalisations to a larger population, however it is clear that the burden placed on the ICU facility by our large HIV+ population is not great. Our unit took part in an earlier study on ICU usage by HIV+ patients in the same region [7]. Whilst it may be difficult to make direct comparisons between the studies, ICU mortality was 33% in each study despite a lower median APACHE II score in the earlier study by Gill et al., 15 versus 21 in this study. Dickson et al. have published the recent experience of another ONE of the ICUs involved in the original study; median APACHE II score was 18 and ICU mortality 23% [14]. The fact that mortality remains the same in our study whereas APACHE II score was higher may mean that, matched for severity of illness, outcome has improved and sicker patients are admitted to ICU during the later period. The median CD4 was 40 during the period 1993–1997 and 128 in this study. This may reflect the increased proportion of patients receiving HAART (24.5% compared with 56%) though other factors cannot be excluded.

Respiratory illness remains the most common reason for ICU admission in the era of HAART, though as a proportion of the total admissions its frequency has dropped from 51.2 % [7] to 37.2%. The rate of admission for pulmonary diagnosis (particularly PCP) is falling in other series but remains a common reason for ICU admission in HIV+ patients [16, 17]. In particular, admission for PCP has fallen, whereas admission for respiratory failure not due to PCP has risen in this study compared with the pre-HAART era. Two patients had complications of liver disease, variceal haemorrhage, and liver failure. Although conclusions cannot be drawn from such small numbers, our only patient with liver failure died on ICU, consistent with the findings of Dickson et al. where liver failure in the context of HIV had a poor ICU prognosis [14]. Liver disease is making an increasing contribution to mortality in HIV+ patients [25].

There were a large number (28%) of admissions for sepsis in this study period. Despite the small population size, this is likely to represent an increase from 10.5% in 1993–1997 [7]. This is consistent with trends in US death certificate reported in the late 1990s showing an increase in deaths from septicaemia in HIV+ patients [26]. ICU mortality from sepsis was 25% in this study, lower than in 1993–1997 (50%).

The use of renal replacement therapy increased from 1.5% of patients in the 1993–1997 period to 26.8% of patients in this cohort, partially (though not fully) accounted for by the increased number of patients with sepsis who had renal replacement therapy. Renal failure is more common in HIV+ patients than other groups and it is associated with an increase in mortality [27]. The increase in use of renal replacement therapy perhaps reflects an underlying trend to treat these patients more aggressively in the light of their improved long-term prognosis.

Interestingly the majority of patients who were admitted to ICU were admitted for reasons directly related to HIV infection (72%). Of those who were not, at least five had medical conditions caused or exacerbated by drug or alcohol misuse (liver failure, two GI bleeds, one case of pancreatitis and deliberate drug overdose). Although not directly HIV related these admissions (12% of all admissions) underline the significant burden of psychiatric co-morbidity occurring in the context of HIV infection.

In our study, HAART use and CD4 count at ITU admission were not related to long-term survival. However, our population is too small to adequately interrogate this difference. There may be other reasons for changes in CD4 count and/or survival over time, such as improvements in nutrition, prophylaxis or other aspects of care. Two larger studies with long followup did detect an influence of HAART on survival [16, 22]. Four of our patients appeared to be failing HAART; a shortcoming of this study is that we did not collect the reasons for this. Median long-term survival in this study was 1008 days, and no patient who survived for more than 2 years after ICU admission died during the study period. Two recent studies have also reported encouraging long-term survival [16, 22].

This study has significant shortcomings. The population is small making it difficult to generalise to larger populations. Data on antiretroviral therapy are incomplete, and duration and type of regime were not collected. Nevertheless, the small size of the population in this study drawn from such a large unit (5000 patients) is indicative of the improved health of HIV+ patients in the era of HAART. Despite its size and retrospective nature, our study adds to the growing body of evidence that ICU care is not futile even in the setting of advanced HIV disease.

## Figures and Tables

**Table 1:** Characteristics of the 43 patients included in this study. The second column is percentage, unless stated otherwise. IQR: interquartile range; ART: antiretroviral therapy; MSM: men who have sex with men.

	Absolute numbers	Percentage (%) or IQR
Median age (IQR)	44	(40–60)
Male	32	(73%)
Race		
White	23	(53.5%)
Black	10	(23.6%)
Asian	4	(9.3%)
Other	2	(4.7%)
Unknown	4	(9.3%)
HIV risk factor		
Heterosexual	15	(34.9%)
MSM	23	(53.5%)
Injection drug use	3	(7%)
Unknown	2	(4.7%)
On ART		
Yes	24	(55.8%)
No	13	(30.2%)
Unknown	6	(14%)
Median CD4 (IQR)	128	(9–627)
Median APACHE II (IQR)	21	(10–38)
AIDS-defining diagnosis at time of ICU admission	19	(44%)

**Table 2:** Primary reasons for ICU admission and outcomes. Numbers are absolute values (percentage). HR: hazard ratio for death in that group. All 95% confidence intervals for hazard ratios overlap 1.

	Number admitted	ICU mortality	HR	Hospital mortality	HR	Mortality at end of followup	HR
Respiratory	16	7 (43.8%)	1.3	11 (68.8%)	1.7	12 (75%)	1.2
GI	4	1 (25%)	0.8	2 (50%)	1	3 (75%)	1.1
Neurological	4	1 (25%)	0.8	2 (50%)	1	3 (75%)	1.1
Sepsis	12	1 (25%)	0.8	4 (33.3%)	0.6	8 (66.7%)	1
Other	7	2 (28.6%)	0.9	3 (42.9%)	0.8	3 (42.9%)	0.6

Total	43	14 (32.5%)		22 (51.2%)		29 (67.4%)	

**Table 3:** Diagnoses resulting in the need for ICU admission among the patients included in this study.

Diagnosis	No.
PCP	6
Pneumonia	5
TB	3
Neurological infection*	2
Septic shock	8
Neutropaenic sepsis^#^	5
Malignancy^¶^	4
GI bleed	2
Pancreatitis	2
Other^§^	6

*Toxoplasmosis and *Pneumococcal* meningitis.

^#^All related to chemotherapy for malignancy; 4 patients had lymphoma and 1 had anal carcinoma.

^¶^Further 9 patients were admitted for another reason but with a background of malignancy.

^§^One patient with pneumothorax, 1 with liver failure, 1 with renal failure, 1 with status epilepticus, 1 with ulcerative colitis, and 1 with drug overdose.
